# Capillary Leakage on Ultrasound in Children with Dengue

**DOI:** 10.3390/children13010089

**Published:** 2026-01-07

**Authors:** María Teresa Gutiérrez-Arcos, Carlos Alberto Velasco-Benítez, Daniela Alejandra Velasco-Suárez

**Affiliations:** 1 Hospital Universitario del Valle, Universidad del Valle, Cali 760026, Colombia; finders@findersgroup.org; 2 Department of Pediatrics, Universidad del Valle, Cali 760026, Colombia; carlos.velasco@correounivalle.edu.co; 3 Gastrohnup Research Group, Universidad del Valle, Cali 760026, Colombia

**Keywords:** dengue, capillary leakage, ultrasound, pediatrics, ascites, pleural effusion

## Abstract

**Background/Objectives**: Dengue is one of the leading causes of morbidity in children in endemic regions. Capillary leakage is the pathophysiological hallmark of severe dengue, and ultrasound has established as a sensitive tool for its early detection. However, evidence in the pediatric population remains limited. The aim of this study was to identify the presence of capillary leakage detected by ultrasound and its associations in children with dengue. **Methods**: Observational/descriptive/cross-sectional/retrospective study conducted in patients between 6 months and 14 years old with confirmed dengue and warning signs or severe dengue, treated at the Hospital Universitario del Valle in Cali, Colombia, between July 2019 and June 2020. Ultrasound examinations were performed and interpreted by radiologists following an institutional standardized protocol. Associations with capillary leakage were evaluated using the chi-square test and their respective OR and 95% CI. **Results**: A total of 132 children were included. Ultrasound capillary leakage was identified in 95.5%, mainly ascites (83.3%), pleural effusion (46.2%), hepatomegaly (40.9%), and vesicular thickening (39.4%). Associated factors were belonging to school/adolescent group (OR = 13.52; 95% CI = 1.41–646.51; *p* = 0.0031), elevated alanine aminotransferase (OR = 11.06; 95% CI = 1.32–94.82; *p* = 0.0007), and aminotransferase levels grades C–D (OR = 6.87; 95% CI = 0.82–54.59; *p* = 0.0110). Thrombocytopenia and hypoalbuminemia were common. Three deaths (0.9%) occurred in the initially confirmed cohort prior to ultrasound-based inclusion, all of whom presented multiple risk factors for capillary leakage. **Conclusions**: In this cohort ultrasound showed high sensitivity for detecting capillary leakage in pediatric dengue and was associated with school-age/adolescents and liver involvement. Its systematic use could improve early identification of severe forms and optimize clinical management in resource-limited settings.

## 1. Introduction

Dengue is a viral disease caused by the DENV, a complex of four distinct serotypes (DENV-1 to DENV-4) [1]. All serotypes can cause acute disease and are transmitted mainly by bites from Aedes aegypti, an anthropophilic mosquito widely distributed in tropical and subtropical regions [2,3].

Dengue is considered one of the most important tropical diseases worldwide, with its incidence increasing more than thirtyfold in recent decades, in parallel with the geographical expansion of the vectors and, therefore, of the virus [4,5]. Dengue epidemics have a significant economic and social impact, imposing high costs on health services, families, and the productive systems of the affected countries [4,6,7].

Approximately 95.0% of dengue cases occur in children under 15 years old, with infants under one year old and children between four and nine years of age being the most likely to develop severe forms of the disease [6,8]. In the endemic regions of Asia and Latin America, including Colombia, about 10.0% of febrile episodes in children under 16 years old correspond to acute dengue infection [5,8]. Of these, about 19.0% of cases in Asia and 11.0% in Latin America require hospitalization [8].

The pathophysiology of dengue is complex and multifactorial [9,10], characterized by systemic involvement and a wide spectrum of clinical manifestations, ranging from asymptomatic cases to severe and unpredictable presentations [2]. The clinical course depends on various factors, such as the host’s immune status, the viral strain, individual genetics, and age [11,12]. Among the most relevant pathophysiological alterations is capillary leak syndrome, which results from endothelial cells being activated by the viral NS1 protein through Toll-like receptor 4 signaling [13,14,15]. This process triggers the release of inflammatory cytokines and alters endothelial permeability and the glycocalyx [2]. The main mediators involved are tumor necrosis factor alpha (TNF-α), interferon gamma (IFN-γ), interleukins IL-2 and IL-8, vascular endothelial growth factor (VEGF), and components of the complement system [15,16]. However, the exact mechanisms that determine the peak vascular permeability during the defervescence phase are not yet fully understood [2].

Increased vascular permeability can manifest in multiple systems, clinically evident from pleural and [17,18] pericardial effusion, ascites, hepatomegaly, splenomegaly, or thickening of the vesicular wall, among other findings [15,17,18,19,20,21,22]. However, these signs often go unnoticed in the early stages of clinical presentation through conventional physical evaluation [15]. In this scenario, capillary leakage is defined as the presence of ultrasound findings compatible with plasma extravasation, such as ascites, pleural effusion, or gallbladder wall thickening [1].

In this context, ultrasound is established as a highly useful noninvasive diagnostic tool to evaluate and manage pediatric patients with dengue virus infection [23,24,25,26]. Its use allows for the early detection of plasma leakage, classification of the severity of clinical presentation, and estimation of the progression risk to severe forms of the disease [25,26]. Although several studies have described ultrasound findings associated with dengue, most have focused on adult populations, while few investigations have characterized capillary leak syndrome in children or identified the risk factors associated with its development [15,27]. Furthermore, much of the available evidence comes from other countries, highlighting the limited information on the clinical and ultrasound behavior of pediatric dengue in Colombia.

Therefore, the objective of this study was to identify the presence of capillary leak syndrome using ultrasound and to explore its possible associations with clinical, laboratory, and imaging variables in pediatric patients with dengue treated at the Hospital Universitario del Valle “Evaristo García” (HUV) in Cali, Colombia.

## 2. Materials and Methods

An observational, descriptive, cross-sectional, and retrospective study was conducted based on a database of pediatric patients between 6 months and 14 years old treated at the HUV in Cali, Colombia, a referral center in the southwestern part of the country. The patients were diagnosed with acute dengue virus infection, confirmed by serological tests (positive NS1 antigen or positive dengue-specific IgM), and presented warning signs or severe dengue according to the World Health Organization classification [28]. The study period was from 1 July 2019 to 30 June 2020.

From this database, the patient’s medical records were compiled, including sociodemographic (age, sex, and origin), clinical (anthropometric measurements, type and severity of infection, symptoms, and need for intensive care unit (ICU) admission), paraclinical (NS1 antigen, IgM and IgG for dengue, aspartate aminotransferase (AST), alanine aminotransferase (ALT), albumin, complete blood count, and coagulation times), imaging (chest X-ray and abdominal ultrasound reports, chest ultrasound, and echocardiogram), and therapeutic variables (administration of albumin and blood products). All ultrasound examinations were performed and interpreted by radiologists from the HUV with training in pediatric ultrasonography, following an institutional standardized protocol to ensure consistency in image acquisition and enhance the reproducibility of the findings.

The information obtained was exported to Stata version 16.0 for statistical analysis. In the univariate analysis, sociodemographic, clinical, paraclinical, imaging, and therapeutic variables were described using measures of central tendency and dispersion (absolute and relative frequencies, means, and standard deviations). Bivariate analysis was performed using the chi-squared (χ^2^) test to explore associations between the presence of capillary leakage and the aforementioned variables. Odds ratios (ORs) were calculated with their respective 95% confidence intervals (95% CIs). Differences were considered statistically significant when the *p*-value was less than 0.05.

This project was endorsed by the Ethics Committee of the HUV in Cali, Colombia, with approval number 065-2019.

## 3. Results

**Figure 1:**
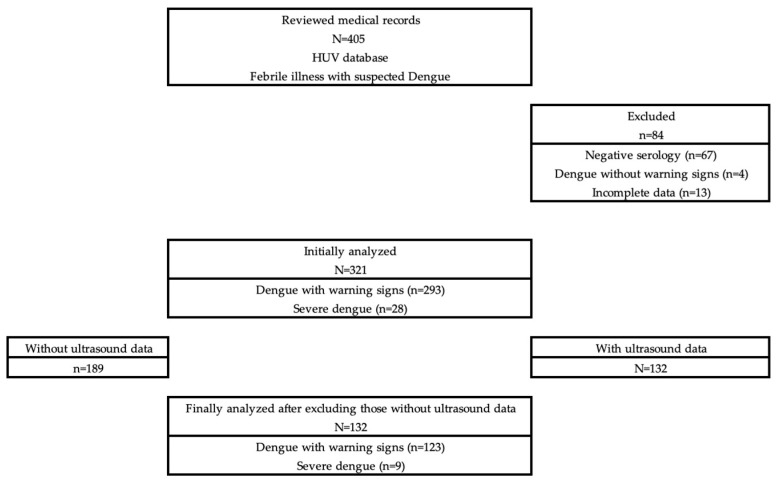
Study flowchart.

A total of 405 children under 14 years old who presented to the Emergency Department of the HUV in Cali, Colombia, with clinical suspicion of acute dengue virus infection were identified. In total, 84 children were excluded: 67 with negative dengue serology, 4 with dengue without warning signs, and 13 with incomplete data. This resulted in 321 children with confirmed dengue and warning signs or severe dengue. Among these 321 patients, 189 were excluded from the main analysis due to the absence of ultrasound data, leaving a final study population of 132 children, including 123 with dengue with warning signs and 9 with severe dengue; this population was included in the ultrasound-based analysis (Figure 1).

### 3.1. General Characteristics

A total of 132 children between 6 months and 14 years old (7.8 ± 3.6 years old, 54.6% schoolchildren) were included, 52.3% of whom were male and 93.9% of whom were from southwestern Colombia. Most had secondary infection, were eutrophic according to the Body Mass Index, and more than half had abdominal pain and vomiting. About one-third of the children had an extended hospital stay lasting more than 5 days, and one-quarter required management in the ICU. More than half of the children had hypoalbuminemia, thrombocytopenia, leukopenia, and grade C aminotransferase levels according to the de Souza classification [29]; 0.8% had prolonged coagulation times, and 6.1% had leukocytosis. Therapeutic management included intravenous albumin and blood products, among other treatments (Table 1).

### 3.2. Imaging

The main finding on chest X-rays was pleural effusion (65.7%). The time elapsed between symptom onset and ultrasound was 6.0 ± 2.1 days, which was considered late (more than 5 days) in 40.1% of cases. In 126 children (95.5%), capillary leakage was evident on ultrasound, involving ascites (83.3%), pleural effusion (46.2%), hepatomegaly (40.9%), a thickened gallbladder (39.4%), and pericardial effusion (3.0%) (Table 2).

### 3.3. Possible Risk Factors

The schoolchildren and adolescent age groups were possibly at higher risk for developing capillary leakage (OR = 13.52, 95% CI = 1.41–646.51, *p* = 0.0031); other risk factors included elevated ALT (OR = 11.06, 95% CI = 1.32–94.82, *p* = 0.0007) and aminotransferase levels grades C + D according to the de Souza classification (OR = 6.87, 95% CI = 0.82–54.59, *p* = 0.0110) (Table 3).

Some associations showed a wide 95% CI, reflecting statistical imprecision due to the small number of patients without ultrasound evidence of capillary leakage. Therefore, these estimates should be interpreted with caution, focusing on the direction of the association rather than the OR magnitude.

### 3.4. Mortality

Three children died (0.9% of the initially confirmed cohort of 321 patients), and due to the severity of their condition, they did not undergo ultrasound examinations. Therefore, they were not included in the final ultrasound-based analysis (N = 132). These cases are described separately to provide contextual evidence highlighting the clinical relevance of the identified risk factors. In all cases, two or more characteristics considered risk factors for capillary leakage were identified according to the criteria established in this study: one patient was a schoolchild, three had elevated ALT, and all three had aminotransferase levels of grades C – D according to the de Souza classification [29] (Table 4).

Although ultrasound findings were not available, the clinical and laboratory characteristic analysis showed that all three patients presented abdominal pain, liver involvement evidenced by elevated AST, hypoalbuminemia, and thrombocytopenia. Two of them had secondary infection, grade D aminotransferase levels according to the de Souza classification [29], severe thrombocytopenia, pleural effusion confirmed by chest X-rays, and required intravenous albumin administration, while the third had grade C aminotransferase levels according to the de Souza classification [29] and mild thrombocytopenia (Table 5).

## 4. Discussion

### 4.1. Prevalence of Capillary Leakage Ultrasound Findings

Our study identified a high prevalence of ultrasound signs of capillary leakage, especially pleural effusion, ascites, and thickening of the vesicular wall, findings that are consistent with the international literature on pediatric populations [15,17,18,19,20,21]. Rather than reflecting only disease severity, this high prevalence underscores the ability of ultrasound to detect subclinical plasma leakage that may not yet be apparent on a physical examination, supporting its role as an early diagnostic tool in pediatric dengue.

In the HUV cohort, more than 90.0% of patients with dengue presented at least one sign of plasma extravasation, which supports the role of ultrasound as a sensitive tool to detect complications in dengue early on. These results are in line with those reported by Foucambert et al. [1], who found signs of capillary leakage in 80.0% of children with severe dengue and demonstrated that vesicular wall thickening > 3 mm is associated with greater severity. In our population, the presence of vesicular thickening in patients with dengue and other warning signs suggests that this finding may represent an early marker of endothelial dysfunction, preceding overt clinical deterioration, reinforcing the value of ultrasound beyond its use in advanced disease stages.

### 4.2. Implementation of Ultrasound Protocols and Serial Assessment

In our cohort, the high frequency of ultrasound-detected capillary leakage, even among children with dengue and other warning signs, supports the AEDES approach of systematic and sequential ultrasound assessment [30], which proposes repeated evaluations to monitor hemodynamic progression. These findings suggest that the early identification of subtle imaging changes, such as mild ascites, minimal pleural effusion, or vesicular wall thickening, may precede clinical deterioration and allow for timely risk stratification, closer monitoring, and optimizing fluid management. Therefore, our results provide clinical evidence that reinforces the applicability of the AEDES protocol in pediatric populations, particularly in referral centers and endemic settings where recognizing severe dengue early on is critical.

Similarly, the meta-analysis conducted by Kaagaard et al. [17] reported an overall prevalence of pleural effusion of 33.0% (95% CI: 29.0–37.0%), higher in children (43.0%) and in severe cases of dengue (48.0%). The higher frequency observed in our cohort (46.2%) likely reflects the referral nature of our institution, which concentrates moderate and severe cases from southwestern Colombia, highlighting how the healthcare setting and evaluation timing may influence ultrasound detection rates.

Parmar [31] previously warned that ultrasound should not be considered an isolated screening method, as its sensitivity depends on the clinical phase of the disease and the operator. This observation is particularly relevant for dengue, where vascular permeability fluctuates dynamically, thus reinforcing the need to interpret ultrasound findings in conjunction with clinical and laboratory parameters rather than as standalone indicators. In our study, ultrasound examinations were performed by trained radiologists using an institutional protocol; however, variability in the timing of image acquisition in relation to the clinical phase of dengue may have influenced the detection of subtle or early manifestations of capillary leakage.

### 4.3. Factors Associated with Capillary Leakage

From a pathophysiological point of view, Qiu et al. [16] demonstrated in murine models that heterologous secondary infections increase capillary permeability through antibody-mediated immunopathological mechanisms. Consistent with this, 76.5% of our population had secondary infection, and this group showed a higher frequency of liver and ultrasound abnormalities, supporting the hypothesis that abnormal immune response plays a decisive role in capillary leakage.

The Colombian study conducted by Osorio et al. [19] found that point-of-care ultrasound (POCUS) detected capillary leakage in 85.1% of patients and was associated with thrombocytopenia and an increased risk of hospitalization, findings that are consistent with our results. Our findings extend this observation by demonstrating laboratory markers with hepatic involvement and hypoalbuminemia parallel imaging evidence of plasma leakage, reinforcing the concept that capillary leakage represents a multisystem process rather than an isolated imaging phenomenon, as previously suggested by Sivasubramanian et al. [14] in adults.

Although nutritional alterations were common, no statistically significant association with capillary leakage was identified. This finding may be related to incomplete anthropometric data and limited sample size rather than the absence of a true biological relationship, especially given emerging evidence suggesting that both malnutrition and obesity may modulate immune and inflammatory responses in dengue [12]. Even so, almost half of the children had some degree of anthropometric abnormality, suggesting that extreme nutritional conditions, such as malnutrition or obesity, could influence the immunometabolic response to the virus and, potentially, the risk of developing capillary leakage.

### 4.4. Clinical Implications, Prognostic Value, and Mortality

Thanh Vo [32] showed that the use of POCUS to guide resuscitation in children with dengue shock reduced mortality from 64.0% to 15.0%. Although our study was retrospective and did not evaluate ultrasound-guided interventions, the strong association between ultrasound findings and recognized risk factors suggests that the early identification of capillary leakage could meaningfully influence clinical decision-making, particularly regarding fluid management and monitoring intensity.

At the national level, the systematic review conducted by Rodríguez-Morales et al. [5] confirms Colombia’s hyperendemic pattern with a predominance of the DENV-2 serotype, which is disproportionately affecting children and adolescents, especially in Valle del Cauca. Our local data fit this epidemiological pattern: almost all cases were in children and adolescents, a group identified as being at higher risk for severe dengue.

Although mortality in our cohort was low, the fact that all fatal cases shared multiple laboratory and clinical risk factors for capillary leakage reinforces the prognostic relevance of these variables, even in the absence of ultrasound data. These findings are consistent with those reported in previous studies, where the combination of liver involvement, hematological abnormalities, and plasma extravasation has been associated with an increased risk of fatal outcomes in pediatric dengue [1,14]. The absence of imaging in these rapidly evolving cases highlights the importance of early bedside assessment and supports the growing role of POCUS in acute pediatric care [19,32]. This aspect is particularly relevant in endemic regions such as Colombia, where the burden of disease and pediatric hospitalization remain high [5]. Collectively, this evidence supports the need to implement structured ultrasound evaluation protocols in emergency departments and hospitals, especially during epidemic outbreaks.

Overall, our findings align with the growing body of evidence indicating that ultrasound, particularly when integrated with clinical and paraclinical data, offers relevant prognostic value in pediatric dengue within hospitalized and clinically selected populations. However, the high frequency of ultrasound-detected capillary leakage observed in this cohort should be interpreted considering a potential selection bias, as ultrasound examinations were preferentially performed in children with warning signs or severe disease. Consequently, variations across studies likely reflect not only differences in epidemiological context, evaluation timing, and healthcare system complexity but also differences in patient selection rather than inconsistencies in ultrasound performance itself.

In addition, note that the outcome “capillary leakage” was defined as a composite variable, based on the presence of any ultrasound finding compatible with plasma extravasation. In this clinically selected cohort, this resulted in a very high prevalence of the outcome, which limited variability and reduced the discriminative capacity of bivariate analyses. Consequently, the observed associations should be interpreted primarily in terms of their direction rather than their magnitude. Future studies may benefit from evaluating predictors of specific ultrasound findings, such as ascites, or from stratifying capillary leakage according to the number of affected compartments to better capture gradients of severity and improve clinical applicability.

Finally, comparative evidence highlights that implementing standardized ultrasound protocols, such as AEDES, and training clinical staff can improve the reproducibility and impact of this tool in pediatric practice. Our results support the need to incorporate systematic ultrasound into the evaluation of pediatric patients with dengue, especially in endemic regions such as southwestern Colombia, where the burden of disease and infant mortality remains high.

### 4.5. Limitations

This study has several limitations. First, the high prevalence of ultrasound-detected capillary leakage likely reflects a selection bias, as ultrasound examinations were preferentially performed in hospitalized children with warning signs or severe dengue, and a substantial number of patients were excluded due to the absence of ultrasound data. Therefore, the prevalence reported should not be extrapolated to unselected pediatric dengue populations or to patients with mild disease. Its retrospective design limited the availability of some variables that were not consistently recorded in the medical records during patient care, which may have resulted in missing data for certain analyses. In addition, the single-center nature of the study may limit the generalizability of the findings to other settings or populations with different epidemiological or healthcare characteristics. Furthermore, some variables showed a wide 95% CI, likely reflecting the limited sample size, suggesting that future studies with larger populations, preferably multicenter in design, are needed to improve the precision of the estimates. Finally, none of the patients who died underwent ultrasound evaluation due to rapid clinical deterioration, highlighting the potential value of bedside ultrasound (POCUS) and supporting the need to incorporate structured ultrasound training into pediatric residency and specialization curricula.

## 5. Conclusions

In this selected pediatric cohort of hospitalized children with dengue and warning signs or severe disease, ultrasound was used to detect a high frequency of capillary leakage, which was associated with clinical and laboratory markers of severity. These findings support the clinical utility of ultrasound as an adjunct tool for early identification and risk stratification in pediatric dengue while acknowledging that prevalence estimates cannot be extrapolated to unselected populations.

## Figures and Tables

**Table 1:** General characteristics of children with Dengue. N = 132.

Sociodemographic Variables	
**Age (years)**	
Mean ± standard deviation (X ± SD)	7.8 ± 3.6
Range	0.6–14
**Age groups (*n*, %)**	
Infants (6 months to 2 years old)	11 (8.3)
Toddlers (>2 to 4 years old)	28 (21.2)
Schoolchildren (5 to ≤12 years old)	72 (54.6)
Adolescents (13 to ≤18 years old)	21 (15.9)
**Gender (*n*, %)**	
Female	63 (47.7)
Male	69 (52.3)
**Origin (*n*, %)**	
Cali	73 (56.2)
Valle del Cauca	49 (37.7)
Outside Valle del Cauca	8 (6.2)
**Clinical variables**	
**Type of infection (*n*, %)**	
Primary	31 (23.5)
Secondary	101 (76.5)
**Nutritional status (*n* = 76)**	
**According to Body Mass Index (*n*, %)**	
Eutrophic	41 (54.0)
Malnourished	35 (46.0)
**Symptoms (*n*, %)**	
Abdominal pain	104 (80.0)
Vomiting	61 (50.0)
Epistaxis	33 (26.6)
Diarrhea	20 (16.7)
**Hospital stay**	
Mean ± standard deviation (X ± SD)	4.1 ± 3.2 days
Prolonged hospital stay (>5 days) (*n*, %)	38 (29.0)
Intensive Care Unit (*n*, %)	33 (25.0)
**Paraclinical variables**	
**Aminotransferases (X ± SD)**	
Aspartate aminotransferase IU/L	271 ± 530
Alanine aminotransferase IU/L	124 ± 142
**Degree of aminotransferaseemia according to de Souza classification (*n*, %)**	
A	0 (0.0)
B	19 (14.4)
C	72 (54.6)
D	41 (31.1)
**Albumin (*n* = 97)**	
Mean ± standard deviation (X ± SD)	3.31 ± 0.59 g/dL
Hypoalbuminemia (<3.5 g/dL) (*n*, %)	55 (56.7)
Mild hypoalbuminemia (3.0–3.5 g/dL) (*n*, %)	27 (27.8)
Moderate hypoalbuminemia (2.5–3.0 g/dL) (*n*, %)	18 (18.5)
Severe hypoalbuminemia (2.0–2.5 g/dL) (*n*, %)	9 (9.2)
Critically low hypoalbuminemia (<2.0 g/dL) (*n*, %)	1 (1.0)
**Coagulation times (*n* = 128)**	
Prothrombin time (X ± SD)	11.2 ± 1.1 s
Partial Thromboplastin Time (X ± SD)	43.4 ± 9.7 s
International Normalized Ratio (X ± SD)	1.02 ± 0.13
Prolonged times (*n*, %)	1 (0.8)
**Platelets**	
Mean ± standard deviation (X ± SD)	73,750 ± 62,514/μL
Thrombocytopenia (<150,000/μL) (*n*, %)	115 (87.1)
Mild thrombocytopenia (100,000–150,000/μL) (*n*, %)	16 (12.1)
Moderate thrombocytopenia (50,000–99,999/μL) (*n*, %)	36 (27.2)
Severe thrombocytopenia (<50,000/μL) (*n*, %)	63 (47.7)
**Leukocytes**	
Mean ± standard deviation (X ± SD)	4903 ± 3008/mm^3^
Leukopenia (<4500/mm^3^) (*n*, %)	72 (54.6)
Leukocytosis (>10,000/mm^3^) (*n*, %)	8 (6.1)
**Therapeutic management (*n*, %)**	
Intravenous albumin	64 (48.5)
Requirement for blood products	4 (3.0)

A: normal aminotransferase levels; B: elevated aminotransferase levels between 1 and 3 times the upper limit of normal; C: elevated aminotransferase levels >3 to 10 times the upper limit of normal; D: elevated aminotransferase levels >10 times the upper limit of normal.

**Table 2:** Ultrasound findings of capillary leakage and types of Dengue. N = 126.

	All	Dengue with Warning Signs	Severe Dengue	*p*
	*n* = 126	*n* = 123 (93.2)	*n* = 9 (6.8)	
Ascites	110 (83.3)	101 (82.1)	9 (100.0)	0.183
Pleural effusion	61 (46.2)	56 (45.5)	5 (55.6)	0.405
Hepatomegaly	54 (40.9)	51 (41.5)	3 (33.3)	0.457
Thickened gallbladder	52 (39.4)	49 (39.8)	3 (33.3)	0.496
Pericardial effusion	4 (3.0)	3 (2.4)	1 (11.1)	0.249

**Table 3:** Factors associated with capillary leak in children with Dengue. N = 132.

	Capillary Leakage		OR	95% CI	*p*
	Yes 126 (95.4)	No 6 (4.6)			
**Age groups**					
Infants	10 (7.9)	1 (16.7)	0.43	0.04–22.39	0.4497
Toddlers	24 (19.1)	4 (66.7)	0.11	0.01–0.89	0.0053
Schoolchildren-Adolescents	92 (73.0)	1 (16.7)	13.52	1.41–646.51	0.0031
**Sex**					
Female	61 (48.4)	2 (33.3)	1.00		0.4700
Male	65 (51.6)	4 (66.7)	0.53	0.04–3.88	
**Origin**					
Cali	69 (55.7)	4 (66.7)	0.62	0.05–4.57	0.5952
Valle del Cauca	47 (37.9)	2 (33.3)	1.22	0.16–13.96	0.8215
Outside Valle del Cauca	8 (6.5)	0 (0.0)	n/a		
**Type of infection**					
Primary	29 (23.0)	2 (33.3)	1.00		0.5602
Secondary	97 (77.0)	4 (66.7)	1.67	0.14–12.30	
**Dengue classification**					
With warning signs	117 (92.8)	6 (100.0)	0.649		
Severe	9 (7.2)	0 (0.0)			
**Symptoms**					
Abdominal pain	100 (80.7)	4 (66.7)	2.08	0.17–15.42	0.4031
Vomiting	59 (50.0)	2 (33.3)	1.00	0.07–14.21	1.00
**Hospital stay**	**4.0 ± 3.2**	**5.5 ± 2.7**	**0.2614**		
Prolonged hospital stay	35 (28.0)	3 (50.0)	0.38	0.05–3.06	0.2461
Intensive Care Unit	32 (25.4)	1 (16.7)	1.70	0.18–82.97	0.6295
**Paraclinical variables**					
Aspartate aminotransferase	109 (86.5)	3 (50.0)	n/a		
Alanine aminotransferase	116 (92.1)	3 (50.0)	11.60	1.32–94.82	0.0007
**Degree of aminotransferaseemia according to de Souza classification**					
A	0 (0.0)	0 (0.0)	n/a		
B	16 (12.7)	3 (50.0)	0.14	0.01–1.20	0.0110
C + D	110 (87.3)	3 (50.0)	6.87	0.82–54.59	0.0110
**Albumin (X ± SD)**	**3.31 ± 0.58**	**3.17 ± 0.74**	**0.6408**		
Hypoalbuminemia	53 (56.7)	2 (50.0)	1.32	0.09–18.95	0.7824
Mild hypoalbuminemia	26 (49.0)	1 (50.0)	1.30	0.06–79.59	0.8334
Moderate hypoalbuminemia	18 (33.9)	0 (0.0)	n/a		
Severe hypoalbuminemia	8 (15.1)	1 (50.0)	0.40	0.01–26.51	0.4626
Critically low hypoalbuminemia	1 (1.9)	0 (0.0)	n/a		
**Platelets (X ± SD)**	**71,666 ± 60,897/μL**	**117,500 ± 85,289/μL**	**0.0793**		
Thrombocytopenia	110 (87.3)	5 (83.3)	1.37	0.02–13.45	0.7768
Mild thrombocytopenia	14 (12.7)	2 (40.0)	0.21	0.02–2.88	0.0848
Moderate thrombocytopenia	34 (30.9)	2 (40.0)	0.67	0.07–8.40	0.6681
Severe thrombocytopenia	62 (56.4)	1 (20.0)	5.16	0.48–258.42	0.1101
**Leukocytes (X ± SD)**	**4881 ± 3046**	**5358 ± 2199**	**0.7058**		
Leukopenia	69 (54.8)	3 (50.0)	1.21	0.15–9.37	0.8190
Leukocytosis	8 (6.4)	0 (0.0)	n/a		
**Imaging variables**					
Pleural effusion on chest X-ray	84 (66.6)	2 (33.3)	4.09	0.55–46.49	0.0879
Time from symptom onset to ultrasound (X ± SD)	6.0 ± 2.0	6.0 ± 2.7	1.0000		
Delay in performing the ultrasound	50 (39.7)	3 (50.0)	1.01	0.08–9.31	0.9915
**Therapeutic management**					
Intravenous albumin	63 (50.0)	1 (16.7)	5.0	0.53–240.12	0.1104

**Table 4:** Risk factors associated with capillary leak in the three children who died from Dengue.

	Patient 1	Patient 2	Patient 3
School-age/adolescent	+	−	−
Alanine aminotransferase	+	+	+
Grade C-D of aminotransferaseemia according to the de Souza classification	+	+	+

**Table 5:** Risk factors associated with specific ultrasound findings for capillary leak in the three children who died from Dengue.

	Patient 1	Patient 2	Patient 3
Secondary infection	+	+	−
Abdominal pain	+	+	+
Alanine aminotransferase	+	+	+
Grade of aminotransferaseemia according to de Souza classification	C	D	D
Hypoalbuminemia	Mild	Mild	Mild
Severe thrombocytopenia	Mild	+	+
Pleural effusion on X-ray	+	−	+
Requirement for intravenous albumin	+	−	+
Requirement for blood products	−	−	−

## Data Availability

The data presented in this study are available on request from the corresponding author. The data are not publicly available due to privacy and ethical restrictions.

## Abbreviations

The following abbreviations are used in this manuscript:

TNF-α	Tumor necrosis factor alpha
IFN-γ	Interferon gamma
IL	Interleukins
VEGF	Vascular endothelial growth factor
HUV	Hospital Universitario del Valle “Evaristo García”
ICU	Intensive Care Unit
AST	Aspartate aminotransferase
ALT	Alanine aminotransferase
χ^2^	Chi-square
OR	Odds Ratio
95% CI	95% confidence intervals
X ± SD	Mean ± standard deviation
POCUS	Point-of-care ultrasound
